# Dose‐Dependent Anti‐Obesity and Protective Effects of Chia ( *Salvia hispanica* L.) Seed Oil Against Hematological and Hepatorenal Dysfunction in High‐Fat Diet–Fed Mice

**DOI:** 10.1002/fsn3.72161

**Published:** 2026-07-28

**Authors:** Sabbya Sachi, Mst. Prianka Jahan, Sree Alok Kumar, Mahmudul Hasan Sikder, Md. Zahorul Islam

**Affiliations:** ^1^ Department of Pharmacology Bangladesh Agricultural University Mymensingh Bangladesh; ^2^ Department of Fisheries Technology Bangladesh Agricultural University Mymensingh Bangladesh

**Keywords:** chia seed oil, dose–response, hepatorenal function, high‐fat diet, lipid profile, obesity

## Abstract

Chia seeds ( *Salvia hispanica* L.) are recognized as a functional food with a nutrient‐dense profile and health‐promoting properties. In the present study, hexane‐extracted chia seed oil was investigated for anti‐obesity, hematoprotective, and hepatorenoprotective potentials in high‐fat diet (HFD)‐induced obese mice. The mice were fed a 15% butter‐fortified HFD to induce obesity, followed by supplementation with three chia seed oil (CSO) doses (200, 400, and 600 mg/kg body weight/day) through custom‐formulated feed. For statistical comparison, three control groups were maintained: normal control, HFD control, and chia control (600 mg/kg). CSO supplementation significantly reduced body mass gain, food efficiency ratio, and visceral fat accumulation. Restoration of hematological parameters occurred in the CSO‐treated groups, with increased RBC, PLT, and LYM counts and elevated Hb, HCT, and PCT levels. Elevated WBC counts and altered RBC and PLT indices (MCV, MCH, RDW‐CV, RDW‐SD, MPV, PDW) were also restored toward normal levels following CSO treatment. An improvement in lipid profiles (TG, TC, LDL‐C, HDL‐C) was observed in the CSO‐treated groups. Furthermore, hepatic (ALT, AST, TBIL, ALP) and renal (SCr, BUN) biomarkers were significantly attenuated following CSO supplementation, approaching physiological levels. Histological evaluation of liver and kidney tissues confirmed restoration of HFD‐induced structural impairments. Nonsignificant variation was found between the normal control and chia control groups for most parameters. The calculated CSO doses correspond to human‐equivalent intake, highlighting their translational relevance. These findings suggest dose‐responsive anti‐obesity effects of CSO, along with protective effects against obesity‐associated hematological disturbances and hepatorenal dysfunction.

## Graphical abstract

Chia seed oil ameliorates obesity, dyslipidemia, and hepatorenal dysfunction in high‐fat diet–fed mice.

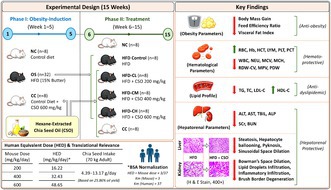

## 1 Introduction

Obesity is one of the most prevalent global epidemics, the occurrence of which has increased notably over the last few decades (Phelps et al. 2024). It is considered a major challenge of the 21st century with significant public health implications (Ahmed and Mohammed 2025). As a potential risk factor, obesity is associated with dyslipidemia, hematological alteration, hypertension, stroke, atherosclerosis, fatty liver, kidney disease, insulin resistance, and cancer (Gutiérrez‐Cuevas et al. 2021; Al‐Jabory et al. 2025). Hence, exploring the optimal approaches to mitigate obesity and its related metabolic complications has been a key concern nowadays (Sadeghi‐Dehsahraei et al. 2024). Conventional strategies to ameliorate obesity comprise maintaining a disciplined lifestyle through dietary regulation and optimized physical activity, along with therapeutic interventions (Kwon et al. 2025). Although orlistat, phentermine, rimonabant, sibutramine, and fluoxetine are considered anti‐obesity drugs, their reciprocal adverse effects (e.g., cardiomyopathy, hypertension, xerostomia, nausea, diarrhea, constipation, dizziness, insomnia, and headache) restrict their widespread use (Li and Cheung 2011). Consequently, the investigation of functional foods to intervene in obesity and associated metabolic disorders with reduced side effects is recognized as a widely adopted alternative approach (Oladimeji and Adebo 2024).

Chia ( *Salvia hispanica* L.), an oilseed of herbaceous plant origin in the Lamiaceae family, is regarded as a functional food attributable to its diverse bioactive components and health benefits (Sachi et al. 2024; Motyka et al. 2023). Chia seed is a plentiful source of bioactive phytochemicals, such as omega‐3 and ‐6 polyunsaturated fatty acids (PUFAs), dietary fibers, and polyphenols (Motyka et al. 2023). It contains various polyphenols, including quercetin, myricetin, rosmarinic acid, kaempferol, and flavonols, as well as omega‐3 PUFAs constituting up to 68%, predominantly alpha‐linolenic acid (ALA) (Knez Hrnčič et al. 2020). These bioactive compounds have been reported to ameliorate oxidative stress, hepatic dysfunction, adiposity, impaired glucose metabolism, insulin resistance, and inflammation associated with obesity‐related metabolic disorders (Enes et al. 2020; de Paula Dias Moreira et al. 2022; Estevam et al. 2026). Moreover, dietary intake of beneficial fats like ALA, particularly in a ratio of 3:1 with linolenic acid as found in chia seeds, is suggestive of preventing fat deposition and metabolic diseases (da Silva et al. 2019). Chia seeds, being a reservoir of omega‐3 PUFAs, especially ALA, and heterogeneous polyphenols, could therefore exert protective properties against adipose deposition, obesity, and obesity‐related metabolic complications. Although chia seeds have been utilized in ethnopharmacotherapy for years, their evidence‐driven benefits still remain obscure in many cases (Mohamed et al. 2019).

Recent studies show that chia seeds confer anti‐inflammatory, antihyperglycemic, cardioprotective, and wound‐healing activities (Paarakh et al. 2025; Pintapagung and Asawapattanakul 2020). Some studies revealed the ameliorative potential of CSO in induced obesity and dyslipidemia (Han et al. 2020), while others found nonsignificant effects (Saadh et al. 2025; Alarcon et al. 2023). Renoprotective activity of chia seed, however, remains poorly understood, with limited reports available so far (Hasan et al. 2024). Hematobiochemical assessment of functional foods is crucial, as hematological parameters are closely linked to biological homeostasis and clinical health status (Carpenter and Maryanovich 2024). However, the hematoprotective effects of CSO remain poorly explored, with inconsistent findings reported across studies (Alarcon et al. 2023; Mihafu et al. 2020). Moreover, no study has evaluated the dose‐associated effects of CSO on visceral fat deposition and hepatorenal functions. According to the published literature, HFD‐fed rodent models are commonly used to investigate obesity‐related chronic disorders (Bastías‐Pérez et al. 2020). Therefore, the current study focused on investigating the dose‐specific anti‐obesity, antidyslipidemic, hematoprotective, and hepatorenal protective effects of CSO in HFD‐induced obese mice. We hypothesized that CSO supplementation would dose‐dependently attenuate HFD‐induced obesity and improve dyslipidemia, hematological alterations, and hepatorenal dysfunction in mice.

## 2 Materials and Methods

### 2.1 Collection of Chia Seed

A registered variety of chia seed ( *Salvia hispanica* L.), BAU‐Chia‐1 (Registration No. 1/10–1/19, June 11, 2019; Ministry of Agriculture, Bangladesh) was used in our present study. The seeds were sourced from the Department of Crop Botany, Bangladesh Agricultural University, and a taxonomist from the same department authenticated the variety.

### 2.2 Processing of Chia Seed for Oil Extraction (CSO)

The seeds were manually cleaned to eliminate impurities and rinsed with distilled water. The seeds were dried at 40°C for 3 days and levigated using an electric grinder. The material was then subjected to maceration in n‐hexane (1:4, w/v) using a partially modified method based on Silva et al. (2016), who reported efficient recovery of CSO while preserving fatty acid composition, including PUFAs such as ALA. The initial macerate and the marc‐squeezed portion were both filtered through Whatman grade‐1 filter paper, and the solvent was removed using a rotary evaporator at 40°C. The resulting CSO was kept in a desiccator overnight to remove residual solvent traces. The yield of CSO was 25.86% (w/w) based on dry seed weight. The extracted CSO was stored at 4°C in an airtight vessel and used for diet preparation within 7 days of extraction. The oil‐formulated diets were prepared in batches, stored in airtight containers in a dark place, and completely consumed within approximately 3 days per batch.

### 2.3 Animals and Diets

Overall, 48 male Swiss albino mice ( *Mus musculus* ), 6 weeks old and weighing 20–24 g, were employed in this study. The mice were obtained from the International Centre for Diarrhoeal Disease Research, Bangladesh (ICDDR,B). The experiment was conducted in the postgraduate laboratory of the Department of Pharmacology at Bangladesh Agricultural University (BAU). The mice underwent a 7‐day acclimation phase before being allocated into different experimental groups. Polycarbonate cages (15 × 9 × 6 in.) fitted with meshed metallic lids were used for housing the mice, with four animals per cage. Animals were reared in a well‐ventilated room with the following environmental conditions: temperature of 27°C± 2°C, relative humidity of 60%–70%, and an approximately 12 h/12 h light/dark cycle maintained through scheduled manual operation of room LED lighting. In the acclimation phase, the mice received standard pellet feed sourced from ICDDR,B and tap water ad libitum. Thereafter, a custom‐formulated pellet diet (Table 1) was provided for the remainder of the study period. The feeds were prepared periodically and stored in separate polyethylene bags at 4°C to safeguard against contamination and spoilage.

**TABLE 1:** Composition of custom‐formulated pellet diet used in HFD‐fed mice.

Ingredients	Amount (g/kg diet)
Wheat flour	691
Lentil powder	200
Soy flour	50
Fish meal	40
Common salt	4
Vitamin and mineral premix	3
Soybean oil	12

*Note:* All ingredients were sourced locally and prepared following standard laboratory formulation.

### 2.4 Formulation of Experimental Diets

The HFD was prepared by supplementing the control feed with 15% (w/w) butter, resulting in an energy content of 471.10 kcal/100 g, compared to 382.79 kcal/100 g in the normal control diet (Table 2). To maintain constant proportions of protein, vitamins, and minerals across all groups, the carbohydrate content in the HFD was reduced by an equivalent amount (15% w/w). This modification resulted in both the normal control (NC) and chia control (CC) diets providing approximately 12 kcal% of total energy from fat sources and 68 kcal% from carbohydrate, whereas the HFDs provided about 41 kcal% from fats and 43 kcal% from carbohydrates. CSO‐mixed feed pellets were prepared by incorporating different doses of CSO into the feed: 200, 400, and 600 mg/kg body weight/day. The amount of CSO added was adjusted based on daily feed intake per mouse, calculated according to body weight throughout the experimental period. During the dark phase (nighttime), feed was restricted to approximately 80% of normal intake to ensure complete consumption of calculated CSO doses in the prepared diets, whereas CSO‐free feed was provided ad libitum during the light phase (daytime). Mice in all groups had free access to water throughout the experimental period. To maintain uniformity in fat content and texture of the feed pellets among the CSO‐treated groups and the HFD group, the equivalent amount of soybean oil in the CSO‐treated groups' feed was replaced with a calculated quantity of chia oil. The proximate composition of the formulated experimental diets is presented in Table 2.

**TABLE 2:** Proximate composition of the experimental diets used in HFD‐fed mice.

Components		NC		HFD		HFD‐CL		HFD‐CM		HFD‐CH		CC	
		g (%)	kcal (%)	g (%)	kcal (%)	g (%)	kcal (%)	g (%)	kcal (%)	g (%)	kcal (%)	g (%)	kcal (%)
Crude fat (%)		5.39	12.67	21.54	41.15	21.89	41.91	21.13	40.53	22.15	42.22	5.28	12.46
Crude protein (%)		18.86	19.71	18.25	15.49	18.02	15.34	18.22	15.53	18.42	15.61	18.16	19.05
Carbohydrate (%)		64.71	67.62	51.06	43.35	50.24	42.75	51.54	43.94	49.77	42.17	65.29	68.49
Crude fiber (%)		5.27	—	4.36	—	4.48	—	4.21	—	4.32	—	4.82	—
Ash (%)		5.78	—	4.80	—	5.37	—	4.90	—	5.34	—	6.45	—
Total energy	kcal/100 g	382.79	100	471.10	100	470.05	100	469.21	100	472.11	100	381.32	100
	kcal/g	3.83		4.71		4.70		4.69		4.72		3.81	

*Note:* CH, 600 mg/kg CSO; CL, 200 mg/kg CSO; CM, 400 mg/kg CSO.

Abbreviations: CC, chia control; HFD, high‐fat diet; NC, normal control.

### 2.5 Ethical Approval

Approval for the study protocol was granted by the Animal Welfare and Experimental Ethics Committee, Bangladesh Agricultural University [Approval No. AWEEC/BAU/2023(2)/23(2b); Date: 09.12.2023], in compliance with the institutional ethical standards for laboratory animal management in research.

### 2.6 Experimental Design

A 15‐week experimental period was structured into two phases: (I) the obesity‐induction phase and (II) the treatment phase, as outlined below.

- Obesity‐Induction Phase (Week 1 to Week 5): Following a 1‐week adaptation period, 48 mice were randomly assigned to three groups using a simple randomization procedure based on a computer‐generated random sequence (Microsoft Excel) as follows:
  - Normal control (NC) (*n* = 8): received a control‐pellet diet.
  - Obese (OS) (*n* = 32): fed a HFD formulated by enriching the control diet with 15% butter (w/w) to induce obesity.
  - Chia control (CC) (*n* = 8): received the control diet supplemented with CSO at 600 mg/kg body weight/day to assess any potential adverse effects of CSO in nonobese mice.

This obesity‐induction phase lasted for 5 weeks, after which the treatment phase commenced.

II Treatment Phase (Week 6 to Week 15): At the end of Week 5, the obese group (OS, *n* = 32) was divided at random (simple randomization) into four treatment groups, each comprising eight mice (*n* = 8), as follows:
  - HFD control: continued on the HFD without treatment.
  - HFD‐CL: received HFD supplemented with CSO at 200 mg/kg body weight/day.
  - HFD‐CM: received HFD supplemented with CSO at 400 mg/kg body weight/day.
  - HFD‐CH: received HFD supplemented with CSO at 600 mg/kg body weight/day.

Hereafter, CSO 200 (CL), CSO 400 (CM), and CSO 600 (CH) will refer to the daily administration of CSO at 200, 400, and 600 mg/kg body weight, respectively. The treatment phase continued for 10 weeks (Weeks 6–15), during which the NC and CC groups were maintained in parallel as nonobese controls. Body weight was measured weekly from Week 1 to Week 15, and feed consumption was monitored on a daily basis throughout the study period.

#### 2.6.1 Dose Justification and Translational Dose Calculation for Humans

The selection of CSO doses was guided by previous studies (Sachi et al. 2024; Grancieri et al. 2022; Mohamed et al. 2022), which reported biological effects in rodents without observed adverse effects. Human‐equivalent doses (HED) were calculated using the body surface area (BSA) normalization method as described by Reagan‐Shaw et al. (2008). The following formula was used to estimate HED:

$$\mathrm{HED}\;\left(\mathrm{mg}/\mathrm{kg}\right)=\text{Mouse dose}\;\left(\mathrm{mg}/\mathrm{kg}\right)\times \left(K_{m}\;\left(\text{mouse}\right)/K_{m}\;\left(\text{human}\right)\right)$$

where *K*_m_ values are 3 for mice and 37 for humans. The resulting HEDs corresponding to mouse doses of 200, 400, and 600 mg/kg/day were 16.22, 32.43, and 48.65 mg/kg/day in humans, respectively.

#### 2.6.2 Collection of Samples

Following the experimental period, the mice were kept fasting for 12 h before anesthesia for sample collection and euthanasia. Anesthesia was induced with ketamine hydrochloride (60 mg/kg body weight, i.p.) and maintained with diethyl ether using the open‐drop method. Following laparotomy, blood samples were drawn from the inferior vena cava using a 3 mL syringe with a 25‐gauge needle. Euthanasia was conducted via exsanguination, which was ascertained by cessation of heartbeat and respiration. The liver, heart, kidneys, and visceral fat were excised for mass determination and histological examination.

#### 2.6.3 Determination of Body Mass Gain, Feed Efficiency Ratio, and Organ Indices

Body mass gain (BMG) and feed efficiency ratio (FER) were determined based on daily feed consumption and weekly body weight data throughout the study period. The weights of the organs (liver, kidneys, and heart) and visceral fat were measured on an electronic balance after rinsing them with physiological saline and blotting dry with filter paper to compute the respective indices. The following formulae were used:

$$\mathrm{BMG}=\text{Terminal body weight}\;\left(g\right)-\text{Baseline body weight}\;\left(g\right),$$

$$\mathrm{FER}\;\left(\%\right)=\left[\mathrm{BMG}\;\left(g\right)/\text{Cumulative feed intake}\;\left(g\right)\right]\times 100,$$

$$\begin{array}{cc} \text{Organ or visceral}\;\mathrm{fat}\;\text{index}\;\left(\%\right)\;=\; & [\text{Organ or visceral}\;\mathrm{fat}\;\text{weight}\;\left(g\right)\\ & /\text{Terminal body weight}\;\left(g\right)]\times 100. \end{array}$$

For the obesity‐induction phase, body weight at the beginning of Week 1 was used as the baseline, and BMG was calculated over Weeks 1–5. For the treatment phase, body weight at the end of Week 5 (i.e., the beginning of Week 6) was used as the baseline, and BMG and FER were calculated over Weeks 6–15.

#### 2.6.4 Blood Sample Processing

Blood samples from each mouse were aliquoted into two portions: one was placed in a 2 mL EDTA (ethylenediaminetetraacetic acid) tube for hematological examination, and the remaining portion was transferred into a 2 mL Eppendorf tube for biochemical analysis. Serum separation was accomplished by centrifuging coagulated blood samples in the Eppendorf tubes at 5000 rpm for 10 min. Blood samples in the EDTA tubes were analyzed immediately after collection, while serum samples were stored at −20°C until biochemical analysis.

#### 2.6.5 Hematological Analysis

Hematological parameters were assessed with an automated hematology analyzer (BHA 560; Benemed Industry Co. Ltd., China). The evaluated parameters included red blood cell (RBC) count, hemoglobin (Hb), hematocrit (HCT), mean corpuscular volume (MCV), mean corpuscular hemoglobin (MCH), mean corpuscular hemoglobin concentration (MCHC), red cell distribution width–coefficient of variation (RDW‐CV), and red cell distribution width–standard deviation (RDW‐SD). Platelet indices, including platelet count (PLT), mean platelet volume (MPV), platelet distribution width (PDW), and plateletcrit (PCT), were also measured. Additionally, white blood cell (WBC) count and differential leukocyte count (DLC), including neutrophils (NEU), lymphocytes (LYM), monocytes (MON), eosinophils (EOS), and basophils (BAS), were determined.

#### 2.6.6 Biochemical Analysis

Serum samples were assessed for biochemical markers, including triglycerides (TG), total cholesterol (TC), high‐density lipoprotein cholesterol (HDL‐C), low‐density lipoprotein cholesterol (LDL‐C), alanine aminotransferase (ALT), aspartate aminotransferase (AST), total bilirubin (TB), alkaline phosphatase (ALP), total plasma protein (TP), albumin (ALB), globulin (GLO), serum creatinine (SCr), and blood urea nitrogen (BUN) using an automated analyzer (Dimension RXL MAX; Siemens, USA) in accordance with the manufacturer's guidelines.

#### 2.6.7 Histological Assessment

Histological processing of liver and kidney tissues was performed following the protocols outlined by Sachi et al. (2024) and Murshed et al. (2024), respectively. Briefly, tissue specimens were preserved in 10% neutral‐buffered formalin, dehydrated through ascending concentrations of ethanol, embedded in paraffin, microtomed at 4–5 μm thickness, and stained with hematoxylin and eosin (H&E) for subsequent examination under a light microscope at 400× magnification. Histological evaluation was performed using a semiquantitative grading method in a blinded manner, analyzing five randomly selected fields per slide. The extent of histopathological alterations was scored as: 0 = normal, 1 = mild, 2 = moderate, and 3 = severe. The steatotic area (%) was quantified using ImageJ software and subsequently converted into the same grading system for consistency (0 = 0%–5%, 1 = 6%–25%, 2 = 26%–50%, 3 = > 50%). The mean score per animal was used for statistical analysis.

### 2.7 Statistical Analysis

All analyses were performed using individual animals as the experimental units. Data are expressed as mean ± standard error of the mean (SEM). Intergroup differences were assessed using one‐way and/or two‐way ANOVA, followed by Tukey's HSD for post hoc analysis. Histological semiquantitative scores were analyzed as ordinal data using the Kruskal–Wallis nonparametric test, followed by Dunn–Bonferroni post hoc comparisons for pairwise analysis. Statistical significance was defined at *p* < 0.05. In all analyses, HFD and CC groups were compared with the NC group, while CSO‐supplemented groups were assessed relative to the HFD control to determine significance.

## 3 Results

### 3.1 Effects of CSO on Body Weight, Organ Weights, Feed Intake, and Feed Efficiency Ratio

**FIGURE 1:**
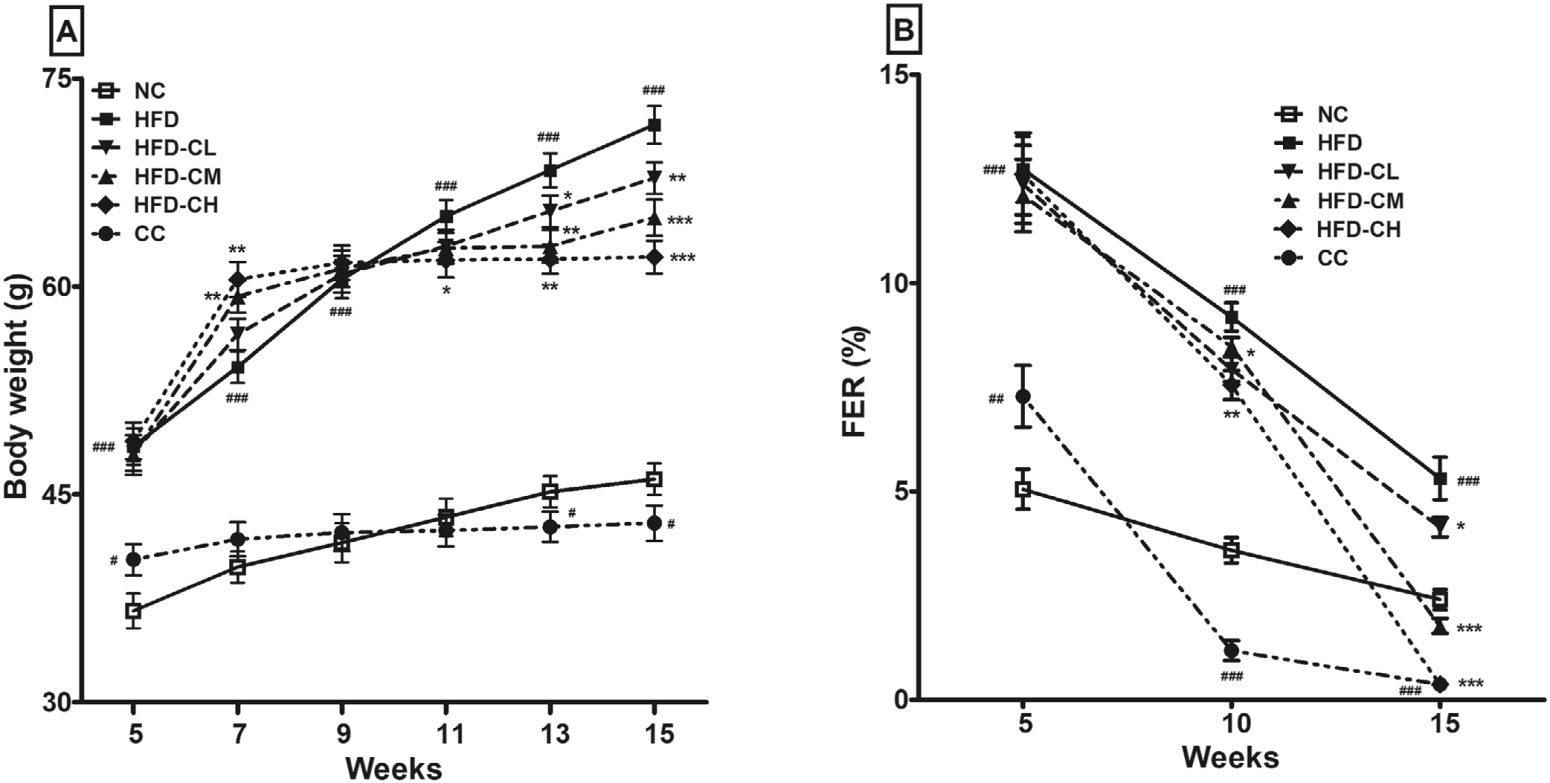
CSO supplementation dose‐dependently reduced body mass gain and FER in HFD‐induced obese mice. Timeline of the effects of CSO on (A) body weight and (B) FER (%). The plotted values represent mean ± SEM (*n* = 8). Intergroup differences were assessed using two‐way ANOVA followed by Tukey's HSD post hoc test. The number of asterisks and hashes indicates the strength of significance versus HFD control (*p < 0.05, **p < 0.01, ***p < 0.001) and the strength of significance versus NC control (^#^ *p* < 0.05, ^##^ *p* < 0.01, ^###^ *p* < 0.001), respectively. CC, chia control; CH, 600 mg/kg CSO; CL, 200 mg/kg CSO; CM, 400 mg/kg CSO; CSO, chia seed oil; FER, feed efficiency ratio; HFD, high‐fat diet; NC, normal control.

**FIGURE 2:**
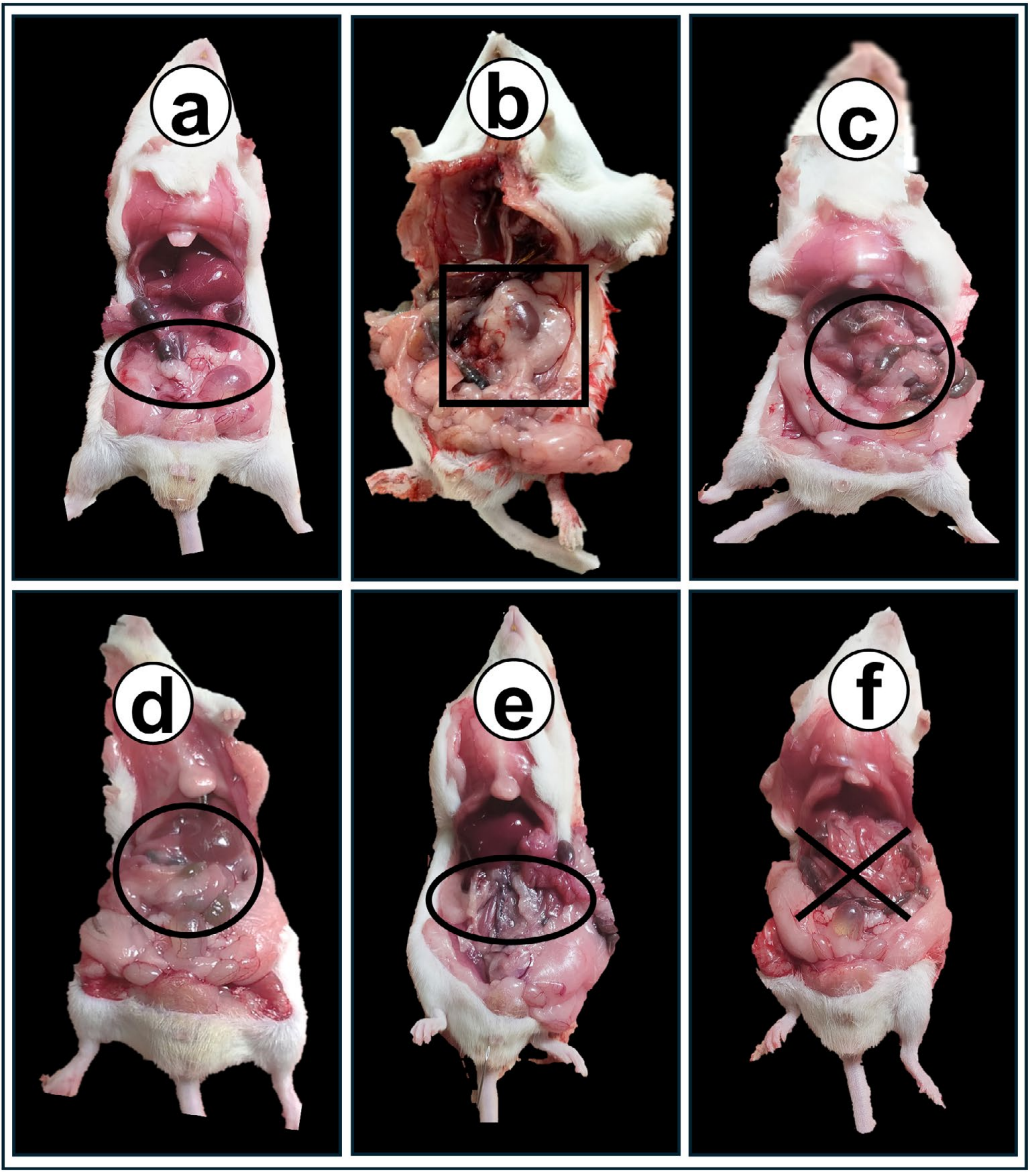
CSO treatment reduced abdominal fat deposition in HFD‐fed mice. Photographic demonstration of the effects of CSO on abdominal fat deposition (a) NC; (b) HFD control; (c) HFD‐CL; (d) HFD‐CM; (e) HFD‐CH; (f) CC. Areas with oval, square, circular, and cross surroundings represent normal, high, moderate, and visibly no abdominal fat, respectively. CC, chia control; CH, 600 mg/kg CSO; CL, 200 mg/kg CSO; CM, 400 mg/kg CSO; CSO, chia seed oil; HFD, high‐fat diet; NC, normal control.

Table 3 outlines the effects of CSO on body mass gain, organ weight parameters, feed intake, and FER. The changes in body weight and FER (%) in a week‐based timeline are demonstrated in Figure 1A,B, respectively. HFD significantly increased body mass gain in the HFD control group by 151.7% relative to the NC during obesity induction (*p* < 0.05), indicating successful establishment of an obesity model for subsequent CSO treatment. The treatment phase also showed a significant elevation in body mass gain and FER (%). Daily feed intake, however, did not vary across groups (Table 3). Treatment with CSO significantly suppressed the body mass gain and FER (%) induced by the butter‐fed diet (*p* < 0.05). Although CSO‐treated groups gained weight until Week 9, the rate slowed over the last 6 weeks dose‐dependently (Figure 1A). Among the CSO‐treated groups, the greatest reduction in body mass gain, 13.31%, was observed in the HFD‐CH group compared with the HFD control group, followed by the HFD‐CM and HFD‐CL groups at 9.35% and 5.39%, respectively (*p* < 0.05). CSO supplementation also caused a substantial fall in FER (%) dose‐dependently (Figure 1B). The HFD control mice had the highest visceral fat index, 6.67% ± 0.26%, which was markedly reduced by CSO treatment dose‐dependently (*p* < 0.05). Attenuation of visceral fat accumulation was highest in HFD‐CH (50.31%), followed by HFD‐CM (37.95%) and HFD‐CL (28.30%) versus HFD control (Table 3). As for the relative weights of the liver, kidney, and heart, no significant differences were observed in the CSO‐treated groups versus the HFD control (*p* > 0.05). Table 3 also demonstrates a significant decline in body mass gain, FER (%), and visceral fat index in the CC group compared with the NC group (*p* < 0.05). Figure 2 illustrates the dose‐dependent effects of CSO treatment on abdominal visceral fat deposition.

**TABLE 3:** Comparative effects of CSO on body weight gain, organ weights, feed intake, and feed efficiency ratio in HFD‐fed mice.

Parameters	Induction period	NC (*n* = 8)	HFD‐induced obese mice (*n* = 32)				CC (*n* = 8)
Body weight (g)	Week 0	28.16 ± 1.08^a^	27.68 ± 1.82^a^				27.95 ± 1.22^a^
	Week 5	36.59 ± 1.24^a^	48.13 ± 1.17^b^				40.29 ± 1.13^a^
Body mass gain (g)	Weeks 0–5	8.36 ± 0.59^a^	21.05 ± 0.24^b^				12.08 ± 0.31^c^
	**Treatment period**	**NC (*n* = 8)**	**HFD (*n* = 8)**	**HFD‐CL (*n* = 8)**	**HFD‐CM (*n* = 8)**	**HFD‐CH (*n* = 8)**	**CC (*n* = 8)**
Body weight (g)	Week 5	36.59 ± 1.24^a^	48.45 ± 1.31^b^	47.98 ± 1.29^b^	47.84 ± 1.44^b^	48.84 ± 1.33^b^	40.29 ± 1.13^b^
	Week 15	46.09 ± 1.13^a^	71.66 ± 1.36^b^	67.80 ± 1.13^c^	64.96 ± 1.29^d^	62.12 ± 1.18^d^	42.92 ± 1.27^e^
Body mass gain (g)	Weeks 5–15	9.62 ± 0.11^a^	23.68 ± 0.88^b^	20.03 ± 0.36^c^	16.94 ± 0.23^d^	13.77 ± 0.18^e^	2.52 ± 0.08^f^
Feed intake/mouse/day (g)		4.77 ± 0.12^a^	4.66 ± 0.25^a^	4.69 ± 0.13^a^	4.76 ± 0.11^a^	4.79 ± 0.09^a^	4.84 ± 0.15^a^
FER (%)		2.88 ± 0.09^a^	7.26 ± 0.16^b^	6.10 ± 0.43^bc^	5.08 ± 0.24^cd^	4.11 ± 0.06^d^	0.74 ± 0.08^e^
Liver weight (g)		2.03 ± 0.23^a^	3.03 ± 0.45^b^	2.78 ± 0.41^bc^	2.62 ± 0.27^c^	2.53 ± 0.17^c^	1.81 ± 0.21^a^
Relative liver weight (%)		4.41 ± 0.15^a^	4.23 ± 0.19^a^	4.11 ± 0.17^a^	4.03 ± 0.11^a^	4.08 ± 0.14^a^	4.21 ± 0.22^a^
Kidney weight (g)		0.22 ± 0.01^a^	0.28 ± 0.02^b^	0.24 ± 0.01^ac^	0.25 ± 0.02^c^	0.25 ± 0.02^c^	0.22 ± 0.01^a^
Relative kidney weight (%)		0.48 ± 0.02^a^	0.39 ± 0.02^b^	0.35 ± 0.01^b^	0.38 ± 0.02^b^	0.41 ± 0.02^b^	0.51 ± 0.03^a^
Heart weight (g)		0.18 ± 0.01^a^	0.31 ± 0.02^b^	0.28 ± 0.02^bc^	0.26 ± 0.01^c^	0.24 ± 0.02^c^	0.16 ± 0.01^a^
Relative heart weight (%)		0.39 ± 0.02^a^	0.43 ± 0.01^b^	0.41 ± 0.02^b^	0.41 ± 0.01^b^	0.39 ± 0.03^b^	0.37 ± 0.02^a^
Visceral fat weight (g)		1.21 ± 0.08^a^	4.77 ± 0.36^b^	3.42 ± 0.17^c^	2.96 ± 0.42^cd^	2.37 ± 0.32^d^	0.74 ± 0.32^e^
Visceral fat index (%)		2.62 ± 0.19^a^	6.67 ± 0.26^b^	5.04 ± 0.27^c^	4.56 ± 0.15^c^	3.82 ± 0.18^d^	1.72 ± 0.08^e^

*Note:* Measurements are presented as mean ± standard error of the mean (SEM) (*n* = 8). Intergroup differences were assessed using one‐way ANOVA, followed by Tukey's HSD for post hoc analysis. Values with dissimilar superscripts within the same row indicate statistically significant differences (*p* < 0.05). CH, 600 mg/kg CSO; CL, 200 mg/kg CSO; CM, 400 mg/kg CSO.

Abbreviations: CC, chia control; HFD, high‐fat diet; NC, normal control.

### 3.2 Influence of CSO on Hematological Indices

Table 4 shows significant decreases in RBC, PLT, and differential LYM counts, Hb, HCT, and PCT in the HFD control group, with significant dose‐related elevations detected in the CSO‐supplemented groups (*p* < 0.05). In contrast, differential NEU count, most RBC indices (MCV, MCH, RDW‐SD, RDW‐CV), platelet indices (MPV, PDW), and WBC count were significantly increased in the HFD group and significantly reduced with CSO treatment (*p* < 0.05). MCHC and other DLCs (MON, EOS, BAS) did not differ significantly across groups (*p* > 0.05). Additionally, no significant variation in hematological parameters was identified between the CC and NC groups (*p* > 0.05).

**TABLE 4:** CSO‐mediated modulation of hematological profiles in HFD‐fed obese mice.

		NC	HFD	HFD‐CL	HFD‐CM	HFD‐CH	CC
RBC (×10^6^/μL)		7.95 ± 0.38^a^	5.24 ± 0.24^b^	5.78 ± 0.53^b^	7.31 ± 0.68^a^	7.28 ± 0.30^a^	8.84 ± 0.76^a^
Hb (g/dL)		13.8 ± 0.16^a^	10.65 ± 0.93^b^	11.82 ± 0.88^b^	13.24 ± 0.52^a^	13.75 ± 0.65^a^	14.13 ± 0.73^a^
HCT (%)		40.75 ± 1.28^a^	31.42 ± 1.44^b^	35.82 ± 1.78^c^	38.23 ± 2.04^cd^	39.16 ± 1.67^ad^	42.11 ± 1.56^a^
MCV (fL)		51.26 ± 2.94^a^	59.95 ± 3.88^b^	61.97 ± 6.47^b^	52.31 ± 5.60^a^	53.79 ± 3.19^a^	47.63 ± 4.46^a^
MCH (pg)		17.36 ± 0.86^a^	20.33 ± 2.09^b^	20.45 ± 2.43^b^	18.11 ± 1.82^a^	18.89 ± 1.16^ab^	15.98 ± 1.60^a^
MCHC (g/dL)		33.86 ± 1.12^a^	33.90 ± 3.49^a^	33.00 ± 2.97^a^	34.64 ± 2.34^a^	35.11 ± 2.24^a^	33.55 ± 2.13^a^
RDW‐CV (%)		13.71 ± 1.27^a^	16.42 ± 1.05^b^	14.57 ± 0.86^ac^	13.92 ± 0.92^a^	13.63 ± 1.15^a^	13.9 ± 0.74^a^
RDW‐SD (fL)		7.03 ± 0.77^a^	9.84 ± 0.89^bc^	9.03 ± 1.07^c^	7.28 ± 0.89^a^	7.33 ± 0.76^a^	6.62 ± 0.71^a^
PLT (×10^3^/μL)		604.67 ± 21.43^a^	374.25 ± 17.22^b^	492.50 ± 15.18^c^	548.17 ± 19.21^d^	559.72 ± 14.87^d^	617.88 ± 18.95^a^
MPV (fL)		6.81 ± 0.54^a^	7.57 ± 0.49^b^	7.05 ± 0.38^b^	6.53 ± 0.29^a^	6.58 ± 0.75^a^	6.31 ± 0.38^a^
PDW (fL)		9.28 ± 1.38^a^	13.03 ± 1.09^b^	12.3 ± 1.21^b^	10.71 ± 1.35^c^	10.12 ± 1.13^ac^	9.18 ± 1.27^a^
PCT (%)		0.41 ± 0.04^a^	0.28 ± 0.02^b^	0.35 ± 0.02^c^	0.36 ± 0.02^c^	0.37 ± 0.04^ac^	0.39 ± 0.02ᵃ
WBC (×10^3^/μL)		6.53 ± 0.94^a^	8.13 ± 0.58^b^	8.05 ± 0.87^b^	7.74 ± 0.74^a^	7.63 ± 0.59^a^	7.24 ± 0.65^a^
DLC (%)	NEU (%)	24.95 ± 2.85^a^	38.78 ± 2.34^b^	36.29 ± 1.96^bc^	34.97 ± 1.89^c^	27.77 ± 0.94^d^	21.84 ± 1.24^ae^
	LYM (%)	71.03 ± 3.42^a^	57.93 ± 2.86^b^	60.43 ± 3.08^bc^	61.52 ± 2.13^c^	68.49 ± 3.54^d^	74.31 ± 2.72^ae^
	MON (%)	1.97 ± 0.85^a^	1.63 ± 0.23^a^	1.68 ± 0.47^a^	1.77 ± 0.12^a^	1.85 ± 0.72^a^	1.88 ± 0.58^a^
	EOS (%)	1.54 ± 0.16^a^	1.18 ± 0.05^a^	1.23 ± 0.22^a^	1.35 ± 0.36^a^	1.47 ± 0.09^a^	1.52 ± 0.14^a^
	BAS (%)	0.51 ± 0.04^a^	0.48 ± 0.08^a^	0.37 ± 0.11^a^	0.39 ± 0.08^a^	0.42 ± 0.13^a^	0.45 ± 0.12^a^

*Note:* All values are expressed as mean ± SEM for each group (*n* = 8). Intergroup differences were assessed using one‐way ANOVA, followed by Tukey's HSD for post hoc analysis. Means with distinct superscript letters within a row denote significant differences (*p* < 0.05). CH, 600 mg/kg CSO; CL, 200 mg/kg CSO; CM, 400 mg/kg CSO.

Abbreviations: CC, chia control; HFD, high‐fat diet; NC, normal control.

### 3.3 Protective Effects of CSO on HFD‐Triggered Dyslipidemia and Hepatic Alterations

#### 3.3.1 Lipid Profile

**FIGURE 3:**
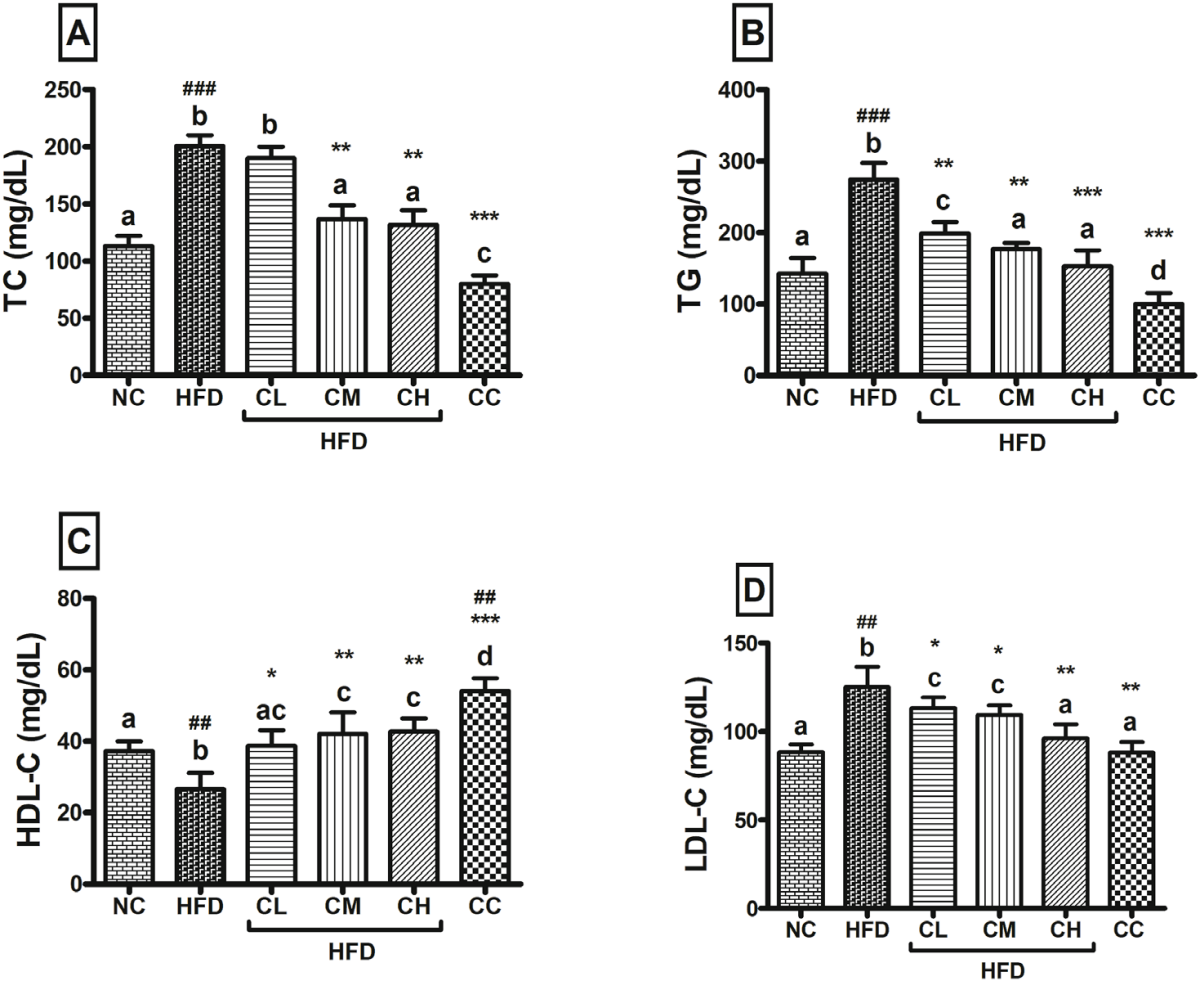
CSO improved the dyslipidemic profile in HFD‐induced obese mice. CSO‐mediated dose‐specific alterations in lipid parameters (A–D). Bar heights depict mean ± SEM for each group (*n* = 8). Intergroup differences were assessed using one‐way ANOVA followed by Tukey's HSD post hoc test. Bars labeled with dissimilar letter codes denote statistical differences among study groups. Asterisks indicate statistically significant differences versus the HFD control (*p < 0.05, **p < 0.01, ***p < 0.001), and hashes reflect significant differences versus the NC group (^#^ *p* < 0.05, ^##^ *p* < 0.01, ^###^ *p* < 0.001). CC, chia control; CH, 600 mg/kg CSO; CL, 200 mg/kg CSO; CM, 400 mg/kg CSO; CSO, chia seed oil; HDL‐C, high‐density lipoprotein cholesterol; HFD, high‐fat diet; LDL‐C, low‐density lipoprotein cholesterol; NC, normal control; TC, total cholesterol; TG, triglycerides.

The effect of CSO on serum lipid profile is shown in Figure 3. CSO treatment markedly improved the HFD‐induced dyslipidemia, with notable reductions in TC, TG, and LDL‐C, and a corresponding rise in HDL‐C (*p* < 0.05). CSO 400 and CSO 600 showed comparable effects on TC, TG, and HDL‐C, whereas differences were observed in LDL‐C. The CC group showed a significantly higher HDL‐C level relative to the NC group (*p* < 0.01).

#### 3.3.2 Hepatic Biomarkers

**FIGURE 4:**
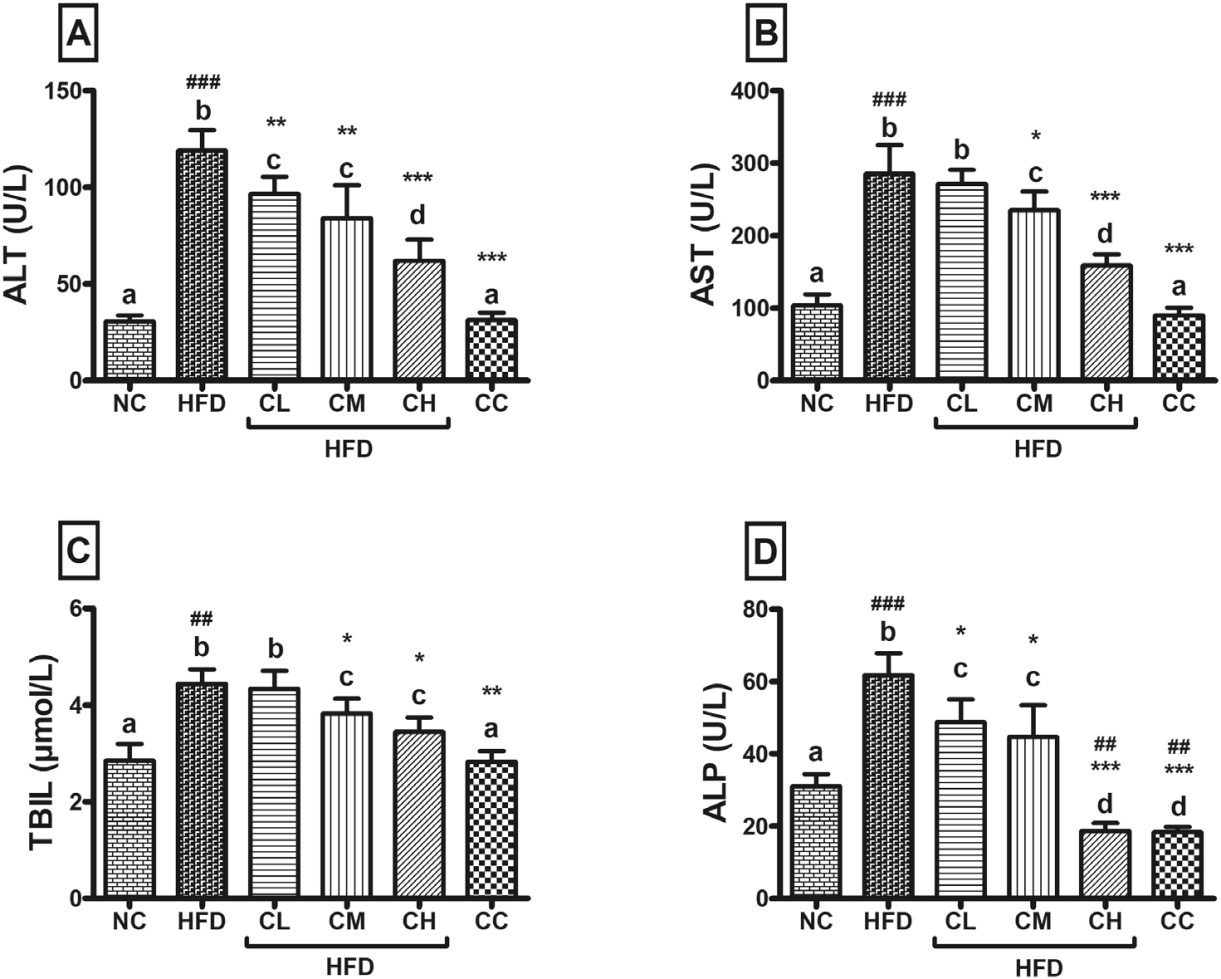
CSO restored HFD‐altered hepatic biomarker levels. CSO‐modulated normalization of HFD‐induced shifts in hepatic biomarkers (A–D). Bar heights depict mean ± SEM for each group (*n* = 8). Intergroup differences were assessed using one‐way ANOVA followed by Tukey's HSD post hoc test. Bars labeled with dissimilar letter codes denote statistical differences among study groups. Asterisks and hashes indicate significant differences versus the HFD control and NC groups, respectively (*p < 0.05, **p < 0.01, ***p < 0.001; ^#^ *p* < 0.05, ^##^ *p* < 0.01, ^###^ *p* < 0.001). ALP, alkaline phosphatase; ALT, alanine aminotransferase; AST, aspartate aminotransferase; CC, chia control; CH, 600 mg/kg CSO; CL, 200 mg/kg CSO; CM, 400 mg/kg CSO; CSO, chia seed oil; HFD, high‐fat diet; NC, normal control; TBIL, total bilirubin.

CSO produced a dose‐dependent improvement in liver‐related biomarkers, as displayed in Figure 4. The elevations in ALT, AST, TBIL, and ALP observed in the HFD control group were significantly attenuated following CSO treatment (*p* < 0.05), with CSO 600 demonstrating the most pronounced efficacy. However, TP, ALB, and GLO levels remained unchanged across groups (*p* > 0.05; Figure 6A). The CC group exhibited a significantly lower ALP concentration (*p* < 0.01), while other markers (ALT, AST, TBIL) did not differ from those of the NC group (*p* > 0.05).

#### 3.3.3 Histopathological Examination of the Liver

**FIGURE 5:**
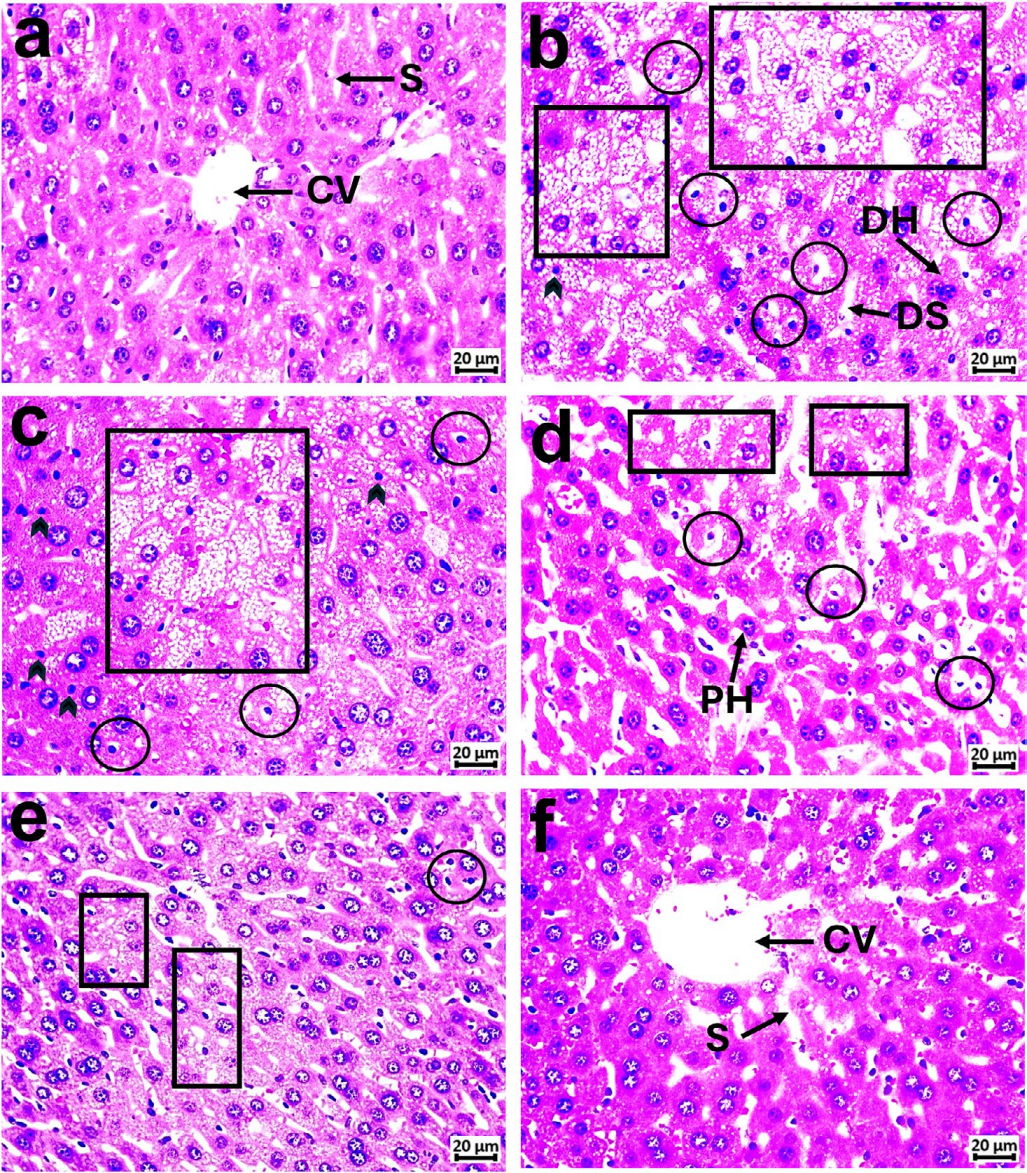
CSO alleviated HFD‐associated alterations in hepatic histopathology (H&E staining). (a) NC; (b) HFD control; (c) HFD‐CL; (d) HFD‐CM; (e) HFD‐CH; (f) CC (microscopic magnification = 400×; scale marker = 20 μm). Arrows highlight the central vein (CV), sinusoids (S), polygonal hepatocytes (PH), degenerated hepatocytes (DH), and dilated sinusoids (DS). Arrowheads denote pyknosis, while areas marked with rectangles and circles represent steatosis and hepatocytic ballooning, respectively. CC, chia control; CH, 600 mg/kg CSO; CL, 200 mg/kg CSO; CM, 400 mg/kg CSO; CSO, chia seed oil; HFD, high‐fat diet; NC, normal control.

NC and CC groups showed standard hepatic morphology (Figure 5a,f). Conversely, the HFD control group presented marked liver parenchymal alterations, including extensive steatosis, hepatocyte ballooning, sinusoidal dilation, and pyknosis (Figure 5b). CSO supplementation conferred noticeable hepatoprotection against hepatic degeneration (Figure 5c–e). Semiquantitative analysis further confirmed significant improvement in HFD‐induced histopathological alterations (Table 5, *p* < 0.05). Although higher doses of CSO showed greater numerical improvement, differences among the treated groups were not consistently statistically significant (Table 5).

**TABLE 5:** Semiquantitative histopathological scores of liver and kidney tissues in HFD‐fed mice.

Parameters		NC	HFD	HFD‐CL	HFD‐CM	HFD‐CH	CC
Liver	Steatosis	0.33 ± 0.07^a^	2.50 ± 0.09^b^	2.00 ± 0.14^b^	0.80 ± 0.07^ab^	0.33 ± 0.07ᵃ	0.27 ± 0.04ᵃ
	Ballooning of hepatocyte	0.53 ± 0.07ᵃ	2.97 ± 0.03^c^	2.70 ± 0.04^bc^	1.03 ± 0.17^abc^	0.83 ± 0.12^ab^	0.57 ± 0.12ᵃ
	Sinusoidal dilation	0.23 ± 0.06ᵃ	2.90 ± 0.10^c^	2.30 ± 0.10^bc^	1.40 ± 0.28^abc^	0.60 ± 0.12^ab^	0.10 ± 0.04^a^
	Pyknosis	0.57 ± 0.14ᵃ	2.60 ± 0.20^b^	1.03 ± 0.06^ab^	0.67 ± 0.12^ab^	0.30 ± 0.09ᵃ	0.30 ± 0.04ᵃ
Kidney	Bowman's space enlargement	0.13 ± 0.07ᵃ	2.60 ± 0.12ᶜ	1.83 ± 0.08^bc^	0.77 ± 0.10^abc^	0.30 ± 0.12^ab^	0.03 ± 0.03ᵃ
	Glomerular capillary dilation	0.03 ± 0.03^a^	1.67 ± 0.11^b^	0.83 ± 0.08^ab^	0.23 ± 0.08^ab^	0.07 ± 0.07^a^	0.03 ± 0.03^a^
	Lipid droplets	0.07 ± 0.04^a^	2.60 ± 0.17^b^	1.93 ± 0.22^ab^	0.27 ± 0.12^ab^	0.03 ± 0.03^a^	0.03 ± 0.03^a^
	Inflammatory cells	0.03 ± 0.03^a^	1.40 ± 0.15^b^	0.47 ± 0.08^ab^	0.17 ± 0.06^ab^	0.07 ± 0.04^a^	0.03 ± 0.03^a^
	Brush border degeneration	0.03 ± 0.03^a^	2.97 ± 0.03^c^	1.60 ± 0.18^bc^	0.87 ± 0.08^abc^	0.40 ± 0.23^ab^	0.10 ± 0.07^a^

*Note:* Values are expressed as mean ± SEM (*n* = 8). Intergroup differences were assessed using the Kruskal–Wallis nonparametric test, followed by Dunn–Bonferroni post hoc comparisons. Different superscript letters within a row indicate significant differences among groups (*p* < 0.05). CH, 600 mg/kg CSO; CL, 200 mg/kg CSO; CM, 400 mg/kg CSO.

Abbreviations: CC, chia control; HFD, high‐fat diet; NC, normal control.

### 3.4 Renoprotective Effects of CSO Against HFD‐Associated Renal Dysfunction

#### 3.4.1 Biochemical Parameters

**FIGURE 6:**
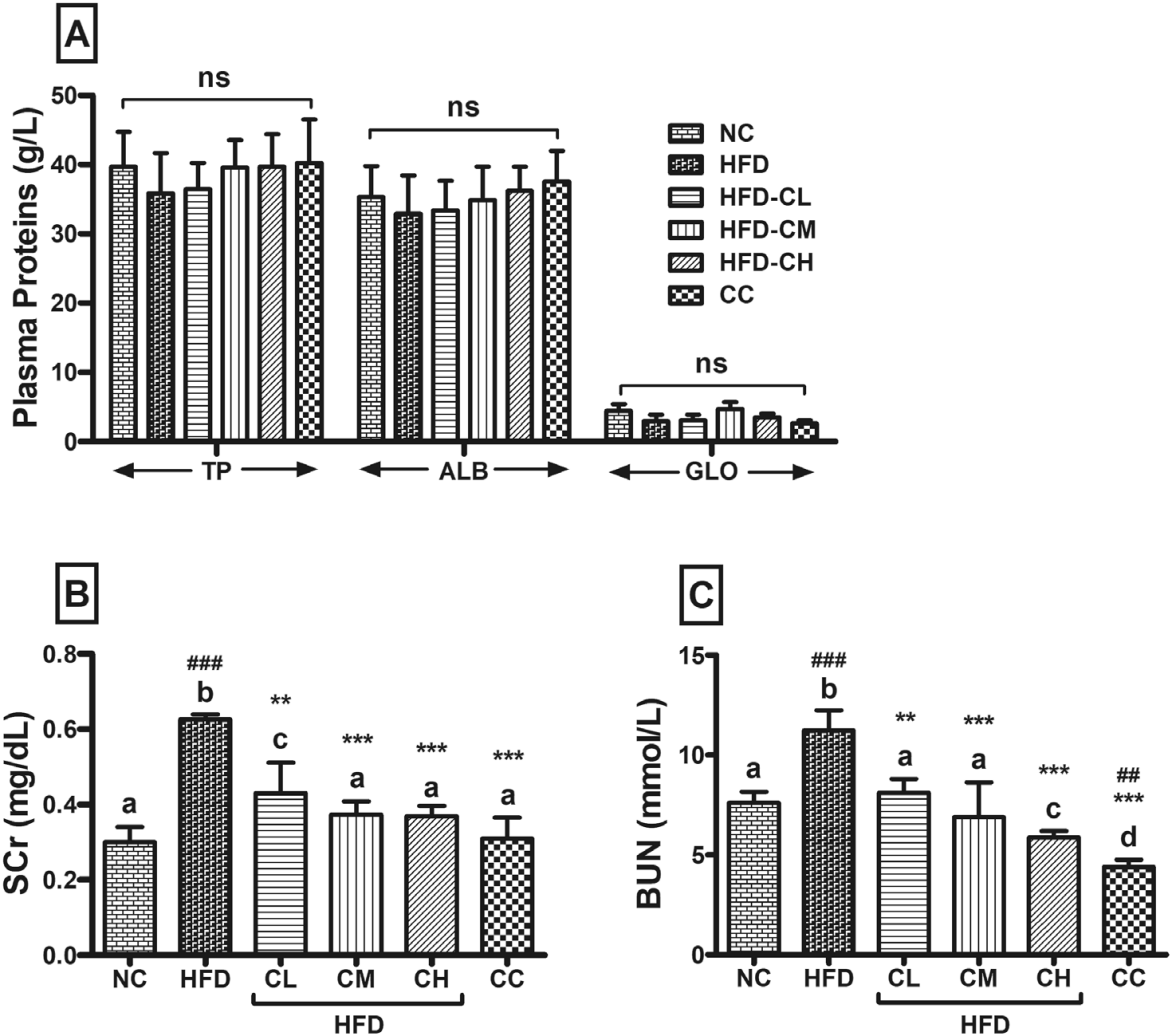
CSO improved renal function biomarkers in HFD‐fed mice without altering plasma protein levels. Roles of HFD and CSO in modulating (A) plasma protein levels and (B, C) renal function markers. Bar heights depict mean ± SEM for each group (*n* = 8). Intergroup differences were assessed using one‐way ANOVA followed by Tukey's HSD post hoc test. Bars labeled with dissimilar letter codes denote statistical differences among study groups, while “ns” denotes nonsignificant variations. Asterisks and hashes indicate significance versus the HFD control and NC group, respectively (*p < 0.05, **p < 0.01, ***p < 0.001; ^#^ *p* < 0.05, ^##^ *p* < 0.01, ^###^ *p* < 0.001). ALB, albumin; BUN, blood urea nitrogen; CC, chia control; CH, 600 mg/kg CSO; CL, 200 mg/kg CSO; CM, 400 mg/kg CSO; CSO, chia seed oil; GLO, globulin; HFD, high‐fat diet; NC, normal control; SCr, serum creatinine; TP, total plasma protein.

Figure 6 illustrates the effect of CSO on kidney function parameters, SCr and BUN. CSO‐supplemented groups exhibited a significant reduction in SCr concentration relative to the HFD control, with no observable differences between CM and CH groups (*p* > 0.05). BUN levels exhibited a substantial decline in the CSO‐treated groups, with the lowest value observed in the CH group (*p* < 0.001).

#### 3.4.2 Histopathological Examination of the Kidney

**FIGURE 7:**
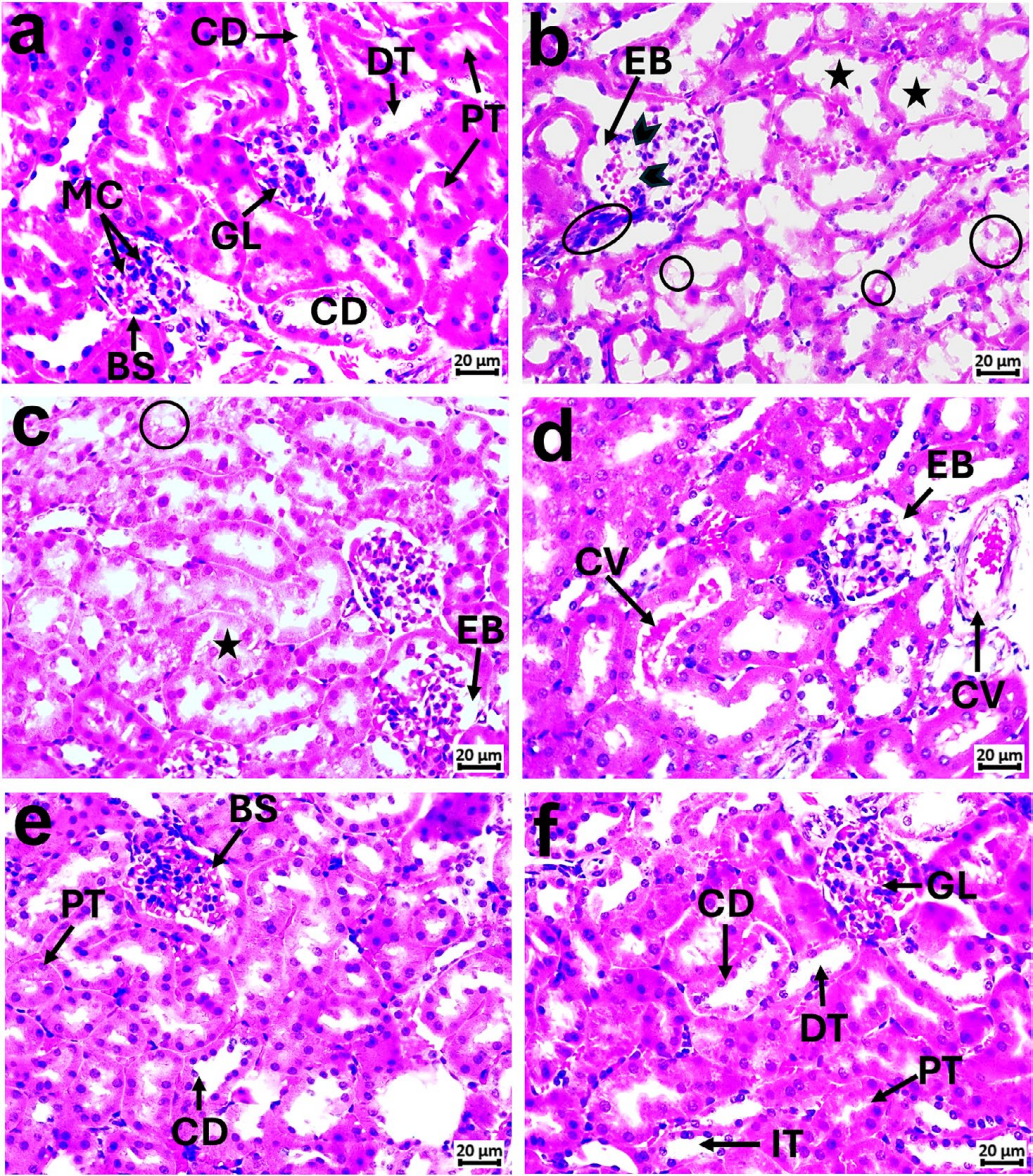
CSO ameliorated HFD‐induced renal histological damage. Kidney histopathology (H&E staining, microscopic magnification = 400×; scale marker = 20 μm) illustrating the protective impact of CSO treatments against HFD‐associated renal damage. (a) NC; (b) HFD control; (c) HFD‐CL; (d) HFD‐CM; (e) HFD‐CH; (f) CC. Arrows indicate the glomerulus (GL), Bowman's space (BS), mesangial cells (MC), proximal tubule (PT), distal tubule (DT), collecting duct (CD), intermediate tubule (IT), enlarged Bowman's space (EB), and congested blood vessel (CV). Areas marked with arrowheads, asterisks, ovals, and circles denote dilated glomerular capillaries, degenerated tubular brush border, mononuclear inflammatory cells, and lipid droplets, respectively. CC, chia control; CH, 600 mg/kg CSO; CL, 200 mg/kg CSO; CM, 400 mg/kg CSO; CSO, chia seed oil; HFD, high‐fat diet; NC, normal control.

Kidney histology of the HFD group revealed abnormalities such as enlarged Bowman's space, dilated glomerular capillaries, infiltration of lipid droplets and mononuclear inflammatory cells, and degeneration of the tubular brush borders (Figure 7b). These structural damages were progressively ameliorated in the CSO‐treated groups (Figure 7c–e). No detectable alterations were observed in the CC group, closely resembling the NC group (Figure 7a,f). Semiquantitative scoring revealed a significant protective effect of CSO against renal injury (Table 5, *p* < 0.05); however, intergroup differences were not consistently statistically significant.

## 4 Discussion

The present findings support our hypothesis that CSO supplementation dose‐dependently attenuates HFD‐induced obesity and ameliorates associated dyslipidemia, hematological alterations, and hepatorenal dysfunction in mice. A significant 1.5‐fold rise in body mass gain in the HFD control group confirmed obesity induction. The higher caloric density of HFD contributed to weight gain by creating an imbalance between caloric intake and expenditure (Maturana et al. 2023). Dietary intake of calorie‐dense saturated fats has been implicated in an increased risk of obesity (Ferreira 2024). Likewise, Miah et al. (2022) reported obesogenic effects of a 10% butter‐enriched diet in mice, consistent with our results using a 15% butter‐enriched HFD. In our study, CSO treatment led to dose‐dependent reductions in body mass gain, accompanied by improvements in feed efficiency ratio (FER %) and visceral fat index (%), in agreement with Munir et al. (2021). This body mass‐lowering effect may be attributable to reduced visceral fat deposition associated with bioactive constituents of CSO, such as omega‐3 PUFAs. Silva et al. (2016) and Ishak et al. (2020) reported that n‐hexane‐extracted chia seed oil contains high levels of omega‐3 PUFAs, including ALA. Among the omega‐3 PUFAs, ALA has been reported to modulate glucose and lipid metabolism and has been linked to the attenuation of visceral adiposity (Ballard et al. 2019). Furthermore, the downregulatory effects of CSO on lipogenic enzymes may limit adipocyte triglyceride storage and fat buildup (Oliva et al. 2021). Despite evidence of fat‐ and weight‐reducing effects of CSO (Mihafu et al. 2020; Fonte‐Faria et al. 2019), the ineffectiveness of chia seed in mitigating body mass gain has also been reported (George et al. 2023), possibly due to differences in experimental setup or treatment duration. A more pronounced rate of body mass gain was observed up to Week 9 across the CSO‐treated groups, followed by a reduced rate of increase in the later phase. This pattern resembles our earlier rat model study, in which chia seed extract produced a similar time‐dependent pattern of body weight change (Sachi et al. 2024). Khatun et al. (2025) also described a comparable time‐dependent effect of chia seed supplementation in broiler chickens. The initial phase of marked weight gain may be linked to antioxidant compounds present in chia seeds that could have counteracted HFD‐induced oxidative stress. These effects may have enhanced digestive enzyme secretion and intestinal motility, thereby improving nutrient absorption and contributing to body mass gain (Alagawany et al. 2020). CSO may also have influenced lipid metabolism throughout the intervention period. The slower rate of body mass gain observed during the later phase may reflect the cumulative reductions in adipose tissue accumulation over time, which is consistent with the observed decreases in visceral fat weight and visceral fat index (%) in CSO‐treated groups at the end of the study. However, the precise mechanisms underlying this temporal pattern remain unclear and require further investigation.

Previous research links HFD intake with hematological alterations caused by oxidative stress and inflammation (Zhang et al. 2022; Ofosu‐Boateng et al. 2024). CSO treatment produced marked improvements in HFD‐induced hematological abnormalities, possibly due to its PUFA content, as PUFAs are known to modulate the hematological profile favorably (Alwan and Hadi 2024). CSO supplementation resulted in significant amelioration of HFD‐associated reductions in RBC and PLT counts, Hb concentration, and HCT (%). Bioactive constituents of CSO may have attenuated lipid peroxidation of the RBC membrane, leading to an enhancement of membrane integrity and resistance to hemolysis. A previous study has shown that ALA's antioxidant properties can enhance erythrocyte membrane stability (Superti and Russo 2024). Furthermore, the hematopoiesis‐modulating effects of antioxidants (Wambi et al. 2008) suggest that CSO phytochemicals could enhance RBC and PLT production via erythropoietin‐like activity or by directly stimulating erythropoiesis and thrombopoiesis. However, further investigations evaluating oxidative stress biomarkers and antioxidant capacity parameters are required to substantiate these potential mechanisms. The increased RBC count in the CSO‐treated groups was accompanied by parallel elevations in Hb and HCT, aligning with the findings outlined by Sachi et al. (2024) and Mihafu et al. (2020). The HFD control group exhibited elevations in RBC indices (MCV, MCH, RDW‐CV, and RDW‐SD) without changes in MCHC levels, indicating normochromic anisocytosis (García‐Escobar et al. 2024; Erhabor et al. 2021). The presence of anisocytosis along with elevated MCV and MCH is frequently associated with vitamin B12 and folate deficiencies, which are characteristic of megaloblastic anemia (Erhabor et al. 2021). These abnormalities may be linked to HFD‐induced impairment of vitamin B12 absorption, potentially caused by gastro‐physiological alterations and changes in the gut microbiome profile (Wan et al. 2022). CSO treatment normalized RBC indices, suggesting a role in reversing HFD‐related normochromic megaloblastic anemia, possibly through restoration of vitamin B12 bioavailability. However, further studies assessing serum vitamin B12 and folate levels are warranted to clarify the mechanisms underlying CSO‐mediated protection. Elevated platelet indices (MPV, PDW) with thrombocytopenia in the HFD control group are suggestive of an inflammatory state (Erhabor et al. 2021). CSO‐elicited revival of platelet indices may reflect its thromboprotective potential. The marked elevation in total WBC count and NEU%, with a corresponding reduction in LYM% in the HFD group, was reversed following CSO treatment. This observation agrees with Zhang et al. (2022), who reported a similar DLC trend in HFD‐fed rats. Obesity‐related inflammatory reactions, accompanied by elevations in IL‐6, IL‐17, TNF‐α, and glucocorticoids, may contribute to lymphocytopenia and enhanced granulopoiesis (Alwan and Hadi 2024). This finding may also be correlated with the mechanism described by Ortega‐Gomez et al. (2022), who demonstrated that inflammatory responses induced by a butter‐enriched HFD trigger neutrophilia in mice through the CXCL2–CXCR2 signaling pathway. Dietary omega‐3 PUFA intake has been reported to be inversely associated with the neutrophil‐to‐lymphocyte ratio (NLR) and WBC count (Li et al. 2024), which may explain the reductions observed following CSO treatment. Similarly, Alarcon et al. (2023) observed that 10% CSO supplementation significantly reduced NLR in rabbits, consistent with the present results. In contrast, Alagawany et al. (2020) noted a significant increase in WBC count in CSO‐treated Japanese quail. This discrepancy may result from species differences and dietary formulation. Hematological evaluation is crucial for assessing the safety margin of xenobiotics, particularly plant extracts (Sarkar et al. 2024). Since CSO at 600 mg/kg in the chia control group caused no significant hematological changes compared with the normal control, this maximum dose can be considered both safe and effective.

HFD consumption resulted in a marked rise in TG, TC, and LDL‐C levels with a concomitant decrease in HDL‐C levels, confirming dyslipidemia. Lipid profile abnormalities may result from the increased fat index (%) in obese mice, reinforcing the involvement of obesity‐related fat accumulation in the emergence of dyslipidemia (Wahabi et al. 2023; Nabila et al. 2023). Moreover, excessive reactive oxygen species (ROS) production in HFD‐induced obesity promotes lipid peroxidation, further contributing to dyslipidemia and related metabolic disorders (Wahabi et al. 2023). The CSO‐led alleviation of HFD‐induced dyslipidemia observed in the current study, particularly at 400 and 600 mg/kg, is in alignment with prior findings (Helal et al. 2023). Mohamed et al. (2022) reported that chia oil at 150 and 300 mg/kg for 30 days improved lipid profiles in hypercholesterolemic rats (Mohamed et al. 2022). The AMPK (adenosine monophosphate‐activated protein kinase)–mediated antiobesogenic properties of chia seed reported by Oliva et al. (2021) may account for its antidyslipidemic potential. Deterioration of the lipid profile is regarded as a key marker of hepatic dysfunction associated with steatosis and metabolic disturbances in obese mice (Wahabi et al. 2023). The present study also revealed hepatic tissue injury, characterized by diffuse hepatosteatosis, ballooning degeneration of hepatocytes, pyknosis, and sinusoidal dilation in the HFD control group. Similar alterations were reported in earlier studies in obese rodent models exposed to HFD (Maturana et al. 2023; Nabila et al. 2023). Aligning with the structural changes, elevated serum concentrations of hepatic biomarkers (ALT, AST, ALP, and TBIL) in the butter‐fed group suggest liver injury associated with long‐term HFD consumption (Wahabi et al. 2023). CSO supplementation markedly alleviated HFD‐induced steatohepatitis and hepatic injury, restoring ALT, AST, TBIL, and ALP levels, which are indicative of CSO's hepatoprotective activity. A previous study has shown that ALA can activate the anti‐lipogenic peroxisome proliferator–activated receptor alpha (PPARα), resulting in the alleviation of hepatic triglyceride accumulation through enhanced hepatic β‐oxidation (Chávez‐Ortega et al. 2024). Additionally, ALA‐induced enhancement of AdipoR2 expression has been reported to activate AMPK in hepatocytes, thereby inhibiting lipid biosynthesis (Nabila et al. 2023). These mechanisms may partly explain the attenuation of hepatic steatosis observed following CSO supplementation. However, further studies evaluating PPARα and related transcription factors are required to elucidate the role of bioactive compounds in CSO within the AdipoR2–AMPK axis. The findings of Helal et al. (2023) are consequently in agreement with ours, showing dose‐dependent improvements in hepatic biomarkers in rats following 28 days of supplementation with chia seed powder. The hepatoprotective effects of CSO are likely attributable to its abundant antioxidant constituents, including omega‐3 PUFAs, phytosterols, and phenolic compounds, which have been reported to mitigate oxidative stress under HFD conditions (Mohamed et al. 2022; Batista et al. 2023). The oxidative stress–mitigating potential of chia oil has been previously described, demonstrating its ability to reduce lipid peroxidation biomarkers, notably malondialdehyde (MDA) and oxidized low‐density lipoprotein (Ox‐LDL) (Mohamed et al. 2022). Moreover, in a study by Batista et al. (2023) on HFD‐fed mice, a 1.5% chia oil–enriched diet for 45 days was observed to enhance hepatic antioxidant defenses, as indicated by elevated activities of glutathione peroxidase (GPx), catalase (CAT), and superoxide dismutase isoforms 1 and 2 (SOD1/2). Thus, the antioxidant properties of CSO may have restored hepatocyte membrane integrity by reducing HFD‐driven lipid peroxidation, consequently preventing the uncontrolled efflux of hepatic enzymes and bilirubin into circulation.

Our results align with prior findings of renal impairments following HFD exposure, evidenced by elevated serum Cr and BUN levels and concomitant histological changes (Mohamed et al. 2022; Wahabi et al. 2023; Sun et al. 2020). Mitochondrial dysfunction and excessive ROS generation caused by HFDs promote lipid accumulation, oxidative damage, and inflammation, collectively contributing to impairment of kidney architecture (Wypych et al. 2023). Sun et al. (2020) noted that 16 weeks of HFD feeding led to higher kidney cholesterol and triglyceride contents, along with elevated ROS production in renal cortical cells. In our study, histoarchitectural analysis revealed enlargement of Bowman's space, dilation of glomerular capillaries, lipid droplet accumulation, mononuclear cell infiltration, and degeneration of tubular brush borders, confirming HFD‐induced renal alterations. Similar patterns of histological orientation were reported in previous studies of HFD‐fed animals, further corroborating our results (Altunkaynak et al. 2008). CSO's role in improving biochemical and histological parameters observed in this study indicates a protective capacity against HFD‐linked renal impairment. Although all CSO treatments showed significant amelioration of biochemical parameters, the corresponding histological scores did not consistently reflect this pattern, suggesting only partial histological protection of kidney tissues in the HFD‐CL and HFD‐CM groups. The renoprotective potential of CSO may stem from its bioactive compounds, including ALA. A 3‐month ALA treatment was found to be effective in protecting renal function in mice in a study by Wypych et al. (2023). In that study, ALA was reported to modulate oxidative stress‐related pathways. In this context, CSO may confer renoprotection by mitigating HFD‐mediated redox imbalance and oxidative stress in kidney tissues. Mohamed et al. (2022) reported significant reductions in serum creatinine and urea levels in hypercholesterolemic rats following supplementation with chia seed oil at doses of 150 and 300 mg/kg body weight, comparable to the renoprotective doses observed in this study. The resemblance in biochemical and histological profiles between the chia control and normal control indicates the safety of CSO at the evaluated doses, consistent with previous observations in rodent models (Sachi et al. 2024). Clinical evidence supports the metabolic benefits of chia seed supplementation at 25–35 g/day in humans, including reductions in body weight and abdominal visceral fat, improvements in lipid profiles, and amelioration of NAFLD‐related metabolic abnormalities (Medina‐Urrutia et al. 2020; Fateh et al. 2024). Nevertheless, a meta‐analysis reported no significant effects on body weight or body fat percentage (TaghipourSheshdeh et al. 2025). The highest CSO dose in this study corresponds to a human‐equivalent intake of ≈3.41 g/day for a 70 kg adult, equivalent to ≈13.17 g of chia seeds based on a 25.86% extraction yield. This is substantially lower than the typical 25–35 g/day supplementation used in human clinical trials (Medina‐Urrutia et al. 2020; Fateh et al. 2024), indicating that daily intake of CSO, even at low experimental doses, is physiologically and nutritionally relevant. These findings highlight the safety and efficacy of CSO at the tested doses (200–600 mg/kg) and their potential translational relevance for humans.

## 5 Limitations of the Study

The fatty acid composition and ALA content of the extracted chia seed oil were not experimentally determined. Therefore, direct associations between the observed effects and specific fatty acid constituents warrant cautious interpretation.

## 6 Conclusion

CSO attenuates HFD‐induced obesity and the associated hematological abnormalities, dyslipidemia, and hepatorenal disturbances, with parallel improvements in histoarchitecture. The restoration of hematobiochemical impairments may be linked to the bioactive constituents present in chia seed oil, including omega‐3 PUFAs, potentially through redox regulation and modulation of lipogenic pathways. Moreover, CSO produces dose‐dependent reductions in body mass gain and exerts protective effects against HFD‐associated complications, with both 400 and 600 mg/kg/day identified as safe and effective doses.

## Author Contributions

**Sabbya Sachi:** writing – review and editing, writing – original draft, visualization, software, project administration, resources, investigation, methodology, formal analysis, funding acquisition, conceptualization, data curation. **Mst. Prianka Jahan:** investigation, writing – review and editing, methodology, data curation, formal analysis. **Sree Alok Kumar:** methodology, investigation, data curation. **Mahmudul Hasan Sikder:** data curation, writing – review and editing, formal analysis. **Md. Zahorul Islam:** writing – review and editing, supervision, visualization, methodology, investigation, formal analysis, conceptualization.

## Funding

Financial assistance for this research was provided by the Ministry of Science and Technology, Government of the People's Republic of Bangladesh, under the Research and Development Project (Project No. 2022/17/MoST (R&D), Period: 2022–2023).

## Ethics Statement

Approval for the study protocol was granted by the Animal Welfare and Experimental Ethics Committee, Bangladesh Agricultural University [Approval No. AWEEC/BAU/2023(2)/23(2b); Date: 09.12.2023], in compliance with the institutional ethical standards for laboratory animal management in research.

## Consent

The authors have nothing to report.

## Conflicts of Interest

The authors declare no conflicts of interest.

## Data Availability

The data that support the findings of this study are available from the corresponding author upon reasonable request.
